# Molecular and clinical effects of aromatase inhibitor therapy on skeletal muscle function in early-stage breast cancer

**DOI:** 10.1038/s41598-024-51751-y

**Published:** 2024-01-10

**Authors:** Tara A. Seibert, Lei Shi, Sandra Althouse, Richard Hoffman, Bryan P. Schneider, Kristen A. Russ, Cody A. Altherr, Stuart J. Warden, Theresa A. Guise, Andrew R. Coggan, Tarah J. Ballinger

**Affiliations:** 1 Indiana University School of Medicine, Indianapolis, IN 46202 USA; 2 Department of Endocrine Neoplasia and Hormonal Disorders, MD Anderson Cancer Center, Houston, TX 77030 USA; 3 Department of Kinesiology, Indiana University School of Health & Human Sciences, Indianapolis, IN 46202 USA; 4 Division of Hematology/Oncology, Department of Medicine, Indiana University School of Medicine, 535 Barnhill Dr. RT 472, Indianapolis, IN 46202 USA; 5 Department of Medical and Molecular Genetics, Indiana University School of Medicine, Indianapolis, IN 46202 USA; 6 Indiana Center for Musculoskeletal Health, Clinical Research Center, Indiana University School of Medicine, Indianapolis, IN 46202 USA; 7 Department of Physical Therapy, Indiana University School of Health & Human Sciences, Indianapolis, IN 46202 USA

**Keywords:** Breast cancer, Cancer microenvironment, Transforming growth factor beta

## Abstract

We evaluated biochemical changes in skeletal muscle of women with breast cancer initiating aromatase inhibitors (AI), including oxidation of ryanodine receptor RyR1 and loss of stabilizing protein calstabin1, and detailed measures of muscle function. Fifteen postmenopausal women with stage I–III breast cancer planning to initiate AI enrolled. Quadriceps muscle biopsy, dual-energy x-ray absorptiometry, isokinetic dynamometry, Short Physical Performance Battery, grip strength, 6-min walk, patient-reported outcomes, and serologic measures of bone turnover were assessed before and after 6 months of AI. Post-AI exposure, oxidation of RyR1 significantly increased (0.23 ± 0.37 vs. 0.88 ± 0.80, p < 0.001) and RyR1-bound calstabin1 significantly decreased (1.69 ± 1.53 vs. 0.74 ± 0.85, p < 0.001), consistent with dysfunctional calcium channels in skeletal muscle. Grip strength significantly decreased at 6 months. No significant differences were seen in isokinetic dynamometry measures of muscle contractility, fatigue resistance, or muscle recovery post-AI exposure. However, there was significant correlation between oxidation of RyR1 with muscle power (r = 0.60, p = 0.02) and muscle fatigue (r = 0.57, p = 0.03). Estrogen deprivation therapy for breast cancer resulted in maladaptive changes in skeletal muscle, consistent with the biochemical signature of dysfunctional RyR1 calcium channels. Future studies will evaluate longer trajectories of muscle function change and include other high bone turnover states, such as bone metastases.

## Introduction

Musculoskeletal toxicity is a significant source of morbidity, noncompliance, and treatment discontinuation in patients taking adjuvant aromatase inhibitor (AI) therapy for estrogen receptor (ER) positive breast cancer, which can ultimately impact survival outcomes^1^. Estrogen deprivation induced by AI therapy contributes to musculoskeletal toxicity by promoting osteoclastic bone resorption and accelerating bone loss, thus predisposing patients to an array of well-established skeletal complications^2^. In addition, preliminary data from preclinical models and observational studies suggests AI-induced bone loss may also contribute to impairments in muscle function, including reductions in grip strength, muscle-specific force, and/or power generation, though relatively little is known about the molecular mechanism(s) underlying these functional impairments^3–6^.

Our preclinical work has elucidated a mechanism implicating changes within the bone microenvironment as a source of muscle dysfunction in high bone turnover states^7,8^. In several different mouse models of osteolytic bone metastases, we discovered that bone destruction secondary to bone metastases results in decreased muscle force production. Increased bone turnover leads to excess resorption and subsequent release of cytokines stored in the mineralized bone matrix, including transforming growth factor-β (TGF-β). TGF-β mediates molecular crosstalk between the skeletal and muscular organ systems by signaling myocytes to upregulate NADPH oxidase 4 (Nox4), resulting in systemic oxidation of skeletal muscle proteins, including the ryanodine receptor/calcium (Ca^2+^) release channel, RyR1^7,8^. RyR1 is located on the sarcoplasmic reticulum and is normally bound to its stabilizing subunit, calstabin1, in a closed state. The RyR1-calstabin1 complex functions as a gatekeeper, sequestering Ca^2+^ in the sarcoplasmic reticulum until signaled via membrane depolarization to dissociate from calstabin1 and destabilize, resulting in massive influx of Ca^2+^ into the myoplasm—a key step in excitation–contraction coupling^9^. In contrast, aberrant destabilization of the RyR1-calstabin1 complex triggered by Nox4-oxidation results in “leaky” RyR1 Ca^2+^ channels, weaker intracellular signaling, and compromised muscle force production^7,8,10^. Furthermore, inhibition along the TGF-β-Nox4-RyR1 pathway attenuates observed impairments in muscle function in preclinical studies, highlighting the potential for therapeutic exploitation of this axis to improve functional outcomes for patients^7^. This biochemical signature of “leaky” RyR1 Ca^2+^ channels—nitrosylation, oxidation, and decreased binding of calstabin1—has been identified in the skeletal muscle of mouse and human models of osteolytic bone metastases and Camurati-Engelmann disease, a non-malignant bone disorder associated with excess TGF-β and muscle weakness^7^. Estrogen deprivation therapy with aromatase inhibition also results in states of bone turnover similar to these models; thus, these findings provide a theoretical basis for the hypothesis that estrogen deprivation induced by AI therapy stimulates osteoclastic bone resorption, secretion of excess TGF-β, and may potentiate muscle dysfunction through oxidation of RyR1 and resultant Ca^2+^ leak in patients with early-stage breast cancer.

Here, we aimed to investigate the impact of AI-induced bone resorption on the skeletal muscle ryanodine receptor RyR1, and to explore the relationship between changes in muscle function at the molecular and clinical levels in women with breast cancer. Establishing the underlying pathophysiology of AI-induced muscle dysfunction will be the obligate first step toward developing mechanistically-based interventions for muscular dysfunction associated with anti-estrogen therapies, thus improving quality of life, compliance, and outcomes for this patient population.

## Materials and methods

### Participant recruitment and eligibility criteria

Fifteen postmenopausal patients with stage I-III ER positive breast cancer planning to initiate an AI were recruited from the Indiana University Melvin and Bren Simon Comprehensive Cancer Center and Eskenazi Health in Indianapolis, IN. Postmenopausal status was defined as age ≥ 60 years, prior bilateral oophorectomy, absence of any menstrual periods in the last 12 months without surgical intervention, or FSH and estradiol in the postmenopausal range. All patients had completed primary therapy for breast cancer including surgery, radiation, and chemotherapy at least 14 days prior to study enrollment. Ongoing trastuzumab and/or pertuzumab therapy was allowed given no known impact on bone turnover or muscle physiology. Additional eligibility included age ≥ 18 years, body weight ≤ 350 lbs. (per dual-energy x-ray absorptiometry (DXA) scan weight limit), and ECOG performance status of 0–1 at the time of study enrollment. Patients with underlying osteoporosis or severe osteopenia (defined as a DXA T-score < 2.0), prior history of a non-traumatic fragility bone fracture, or other disorders affecting bone function or turnover were excluded from the study to more directly evaluate the effect of AI on bone turnover. Past history of vitamin D deficiency was allowed, though current deficiencies required correction to ≥ 20 ng/ml prior to study enrollment; those with vitamin D deficiencies refractory to supplementation were ineligible. Patients taking medications affecting bone metabolism, including bisphosphonates or denosumab, were also excluded.

### Study design

This was a prospective, observational pilot study. Treatment with anastrozole (1 mg) once daily was initiated on day 1 and continued for the duration of the study. Anastrozole was chosen as the initial AI to minimize variability and because it was the most prescribed AI at our institution; however, any AI was allowed as there is no difference in mechanism or impact on bone turnover^11,12^. Patients underwent assessments at baseline prior to starting AI and after 6 months of AI exposure. Assessments included a quadriceps muscle biopsy, DXA measures of body composition and bone mineral density (BMD), isokinetic dynamometry, Short Physical Performance Battery (SPPB), grip strength, 6-min walk test, patient-reported outcome (PRO) questionnaires, and serum samples, as detailed below. Patients were compensated for their time to complete the assessments. The study was approved by the Institutional Review Board (IRB) at Indiana University and performed in accordance with the ethical standards of the Declaration of Helsinki. All patients provided written informed consent.

Patients were contacted monthly for AI dosing information and an estimate of how many doses they missed per week on average. Adherence to AI therapy for the 14 days preceding muscle tissue biopsy collection was required; if on a treatment break during this timeframe, biopsies were delayed until 14 days after AI therapy was restarted. For AI-related toxicities, therapy could be held for ≤ 28 days. Dose adjustments were not permitted. If toxicity occurred, switching to an alternate AI was permitted within 28 days. If the AI was held for > 28 days or discontinued, the patient was removed from the study and not replaced. Adverse events were graded according to the NCI Common Toxicity Criteria (Version 4.0), documented, and reported to the Data Safety Monitoring Committee and/or IRB per study protocol.

### Data collection

#### Demographics

Patient demographics and clinical data, including date of initial diagnosis, prior breast cancer therapies, disease stage, concomitant medications, height/weight, and ECOG performance status, were recorded at baseline.

#### Tissue collection and processing

Muscle biopsies were performed at baseline and 6 months by a physician trained in the procedure (TB). Samples were obtained from the vastus lateralis using the modified Bergström technique as previously described^13^. Muscle tissue samples were immediately flash frozen and stored at – 80 °C. RyR1 was immunoprecipitated from the muscle lysate with 2 µg anti-RyR specific antibody (Santa Cruz Biotechnology, sc-376507) in 0.5 mL of a modified RIPA buffer (20 mM Tris–HCl (pH 7.5), 250 mM NaCl,1 mM EDTA, 1% NP-40, 1 mM Na_3_VO_4_, and Protease Inhibitor Cocktail (Cell Signaling Technology, #5871S) for 4 h at 4 °C. The immune complexes were incubated with Protein G Sepharose^®^ 4 Fast Flow (GE Healthcare, #17-0618-01) overnight at 4°C, and the beads were washed three times with RIPA buffer. The immuno-precipitates were size-fractionated on 4–20% SDS-PAGE gels for RYR1 and 15% for calstabin1, and transferred onto PVDF membranes for 2.5 h at 200 mA. Immunoblots were developed using the following primary antibodies: anti-RyR and anti-calstabin1 (Santa Cruz Biotechnology, sc-133067). To determine channel oxidation, the carbonyl groups in the protein side chains were derivatized to 2,4-dinitrophenylhydrazone (DNP-hydrazone) by reaction with 2,4-dinitrophenylhydrazine (DNPH). The DNP signal was determined using a specific anti-DNP antibody (Millipore, MAB2223). All immunoblots were developed using the SuperSignal West Pico PLUS Chemiluminescent Substrate (Thermo Fisher Scientific, 34577) and detected using an Odyssey system (LI-COR, Inc.). The bait protein RyR1 acts as an internal control. Relative band intensity was quantified using ImageJ Software (NIH).

#### Body composition

Height (to nearest 0.1 cm) and mass (to nearest 0.1 kg) were measured without shoes using a calibrated stadiometer (Seca 264; Seca GmbH & Co., Hamburg, Germany) and scale (MS140-300; Brecknell, Fairmont, MN), respectively. Body mass index (BMI; kg/m^2^) was calculated as body mass relative to height squared. DXA (Norland Elite; Norland at Swissray, Fort Atkinson, WI) was performed to assess BMD and body composition measures (total body fat percentage, total lean mass, and total fat mass). The latter allowed for normalization of strength assessments to total and lean body mass, as previously described^14–16^. Regional DXA scans were performed to obtain femoral neck and lumbar spine BMD and associated T-scores.

#### Muscle performance

Muscle contractile properties, fatigue resistance, and functional recovery of the knee extensors were assessed with a Biodex 4 isokinetic dynamometer (Biodex Medical Systems, Shirley, NY), as previously described^17,18^. Patients performed 3–4 maximal knee extension exercises with their dominant leg at angular velocities of 0, 1.57, 3.14, 4.71, and 6.28 rad/s. The maximal torque, or voluntary force production, generated at each velocity was recorded with 2-min rest periods between sets of contractions. To eliminate artifacts, data was “windowed” to isolate the isokinetic phase and smoothed using a 9-point weighted moving average filter using the manufacturer’s software. Peak power was calculated using the highest achieved torque at each velocity and the resulting power-velocity curve was fit with a parabolic function to determine maximal torque, speed, and power. Subsequently, patients underwent an “all out” 50 contraction fatigue test at 3.14 rad/s to evaluate fatigue resistance during repetitive, maximal activation, measured as a fatigue index comparing power in the first third of contractions to power in the last third. Recovery of muscle function was assessed by measuring restoration of torque during knee extensions performed periodically over the next 10 min. The half-life of torque recovery was calculated by fitting a monoexponential function to the latter data.

Dominant hand grip strength (Jamar Plus + digital hand dynamometer; Sammons Preston, Bolingbrook, IL) and the time taken to complete 5 chair stands were assessed, as we have previously described^19^. Dynamometer performance was confirmed weekly by applying known masses. In addition to raw values, grip strength and repeat chair stand outcomes were converted to age- and sex-matched z-scores relative to our published reference data^19^. Time to walk 4-m from a stationary start at normal speed (usual gait speed) was measured with a stopwatch and converted to speed (m/s). Results from the repeat chair stand, usual gait speed, and a static balance test (ability to balance for 10 s with feet in side-by-side, semi-tandem, and tandem positions) were used to calculate the Short Physical Performance Battery (SPPB) score^20^. A higher score out of 12 indicates better performance. Distance walked in 6 min was measured on a 20-m course.

#### Patient-reported outcomes

Patients completed the physical function domain of NIH Patient-Reported Outcomes Measurement Information System (PROMIS) computerized adaptive test (CAT) (PROMIS-CAT) (version 1.2) to provide a self-reported indication of functional health. PROMIS scores are standardized and expressed as T-scores with a population mean of 50 and standard deviation (SD) of 10^21^.

#### Bone turnover

Serum samples were collected to measure markers of bone turnover. ELISA assays were performed for quantification of N-terminal crosslinked telopeptide of type 1 collagen (NTX1; Novus Biologicals) and TGF-β (isoform TGF-β1) using platelet-free plasma (R&D systems). ELISA was done in triplicate and results were quantified and averaged.

### Statistical analysis

Categorical variables were reported as frequencies and percentages of the total enrolled population. Continuous variables were reported as mean ± SD. The primary endpoint was to compare the relative levels of calstabin1 bound to RyR1 channels in skeletal muscle pre- and post-initiation of AI therapy, measured by coimmunoprecipitation. Ratios of calstabin1to RyR1 were compared at baseline and 6 months post-AI exposure using paired t-tests, or Wilcoxon signed-rank tests if assumptions of paired t-tests were not met. A p value of < 0.05 was considered significant. Compliance with AI therapy, defined as self-report of an average ≥ 80% of daily doses on monthly nursing calls, and participation in baseline and 6-month procedures were required to be evaluable for the primary endpoint. It was estimated at least 12 patients would be needed for feasibility and precision around estimates pre- and post-AI exposure in this pilot study^22^. It was estimated at least 20% of patients would discontinue their AI in the 6 months of study duration and thus 15 patients were enrolled.

Secondary endpoints and analyses included a comparison of changes in relative RyR1 oxidation levels pre- and post-AI exposure using paired t-tests or Wilcoxon signed-rank tests. Differences from baseline to 6-month follow-up in clinical muscle measures, including maximal torque, muscle recovery half-life, total SPPB score, and distance in the 6-min walk test, were compared using paired t-tests or Wilcoxon signed-rank tests. Correlation between changes in RyR1 biochemistry and changes in muscle function were assessed using Pearson correlation coefficients.

## Results

### Patient demographics and clinical data

**Figure 1:**
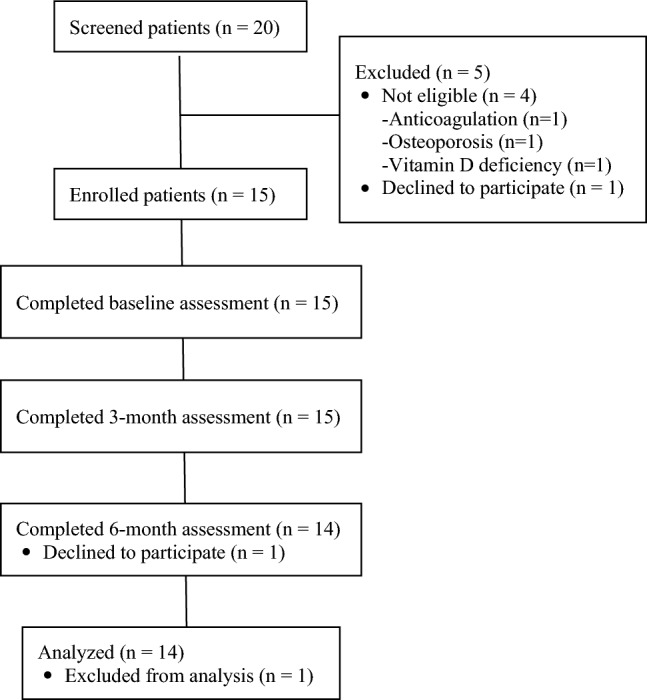
CONSORT.

Fifteen eligible patients (mean age ± SD = 59.9 ± 4.6 years) were enrolled (CONSORT, Fig. 1). Eleven identified as White race. All patients had early-stage breast cancer: 46.6% (n = 7) stage I, 33.3% (n = 5) stage II, and 20% (n = 3) stage III. Three of the patients had previously received chemotherapy and all had received radiation. All patients were initially treated with anastrozole. Four patients experienced AI toxicity of joint pain necessitating a treatment change to an alternate AI (letrozole-3, exemestane-1). All patients reported compliance with AI therapy.

### RyR1 complexes and oxidation in muscle tissue after AI exposure

**Figure 2:**
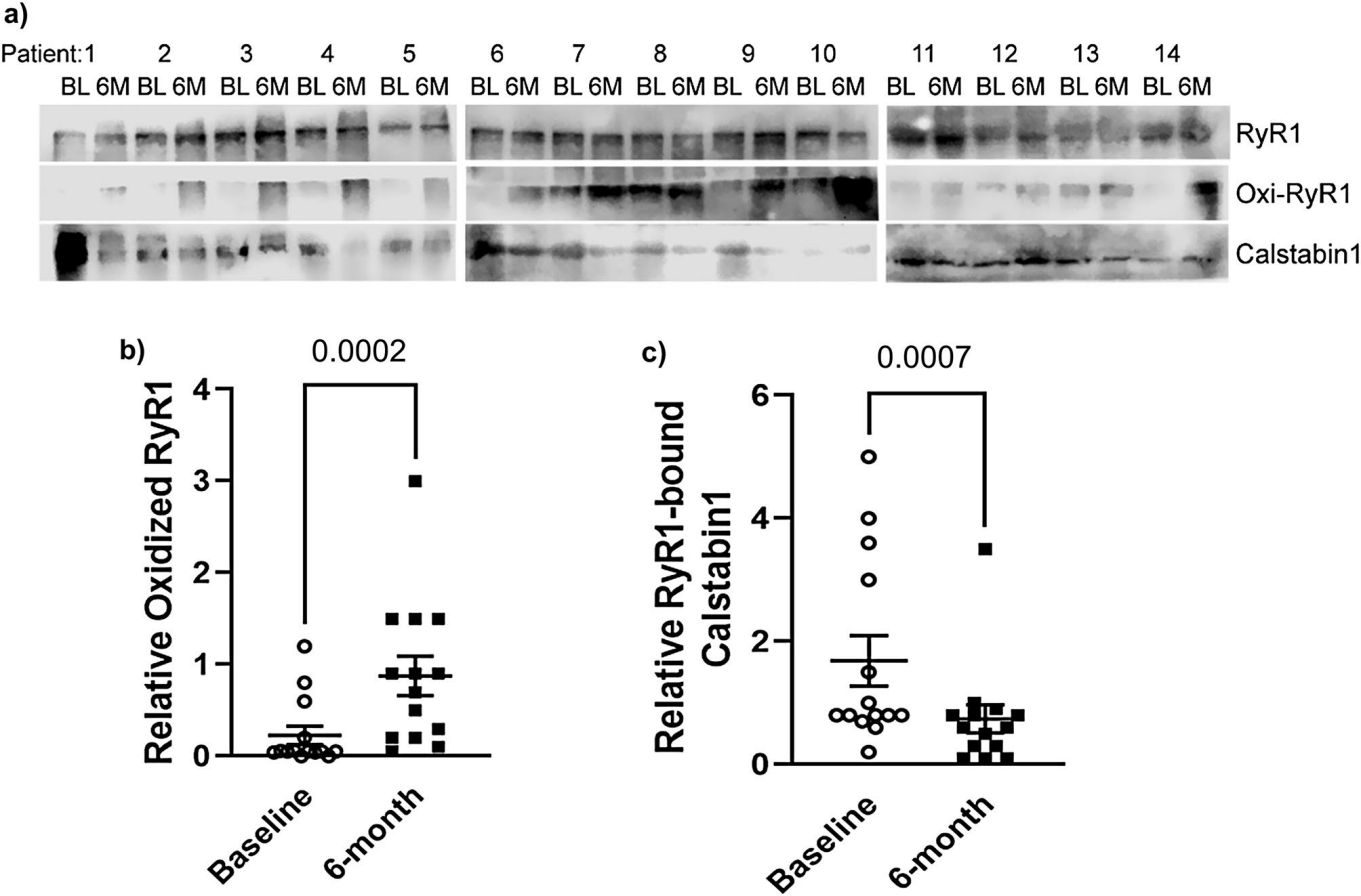
(**a**) Representative immunoprecipitation-Western blot analysis for RyR1 with oxidized thiol groups and their matched total content in homogenate (upper panels) and interaction between RyR1 and calstabin1 (lower panels). BL—baseline; 6 M—6 month follow up. (**b**,**c**) Quantification of relative oxidized RyR1 and the RyR-calstabin1. Results are expressed as mean with SEM. Wilcoxon signed-rank test.

One patient opted to not proceed with the 6-month muscle biopsy and thus 14 patients were evaluable for this endpoint. No adverse events were reported as a result of the biopsy procedure. There was a 2.8-fold increase in oxidation of RyR1 channels after AI exposure between baseline and 6-month follow-up (0.23 ± 0.37 vs. 0.88 ± 0.80, p < 0.001). In addition, there was a more than 50% decrease in bound calstabin1 to RyR1 after AI exposure (1.69 ± 1.53 vs. 0.74 ± 0.85, p < 0.001), consistent with a biochemical signature of dysfunctional and leaking Ca^2+^ channels (Fig. 2).

### Muscle contractile properties and physical function after AI exposure

For those properties with established normative values, patients had normal hand grip strength (z-score = 0.28; 95% confidence interval [CI], − 0.30 to 0.86) and time to complete 5 chair stands (z-score = − 0.14; 95% CI − 0.57 to 0.28) at baseline. Their self-reported functional health was 0.32 SD (95% CI 0.03 to 0.60 SD) below normal, as assessed via the physical function domain of the PROMIS-CAT.

There were no significant differences between baseline and 6-month follow-up in knee extensor muscle power, fatigue index after 50 “all out” contractions, or time to muscle recovery (Table 1). There was a 10.5% (95% CI 3.7% to 17.3%) decrease in grip strength over 6 months (26.2 ± 5.9 kg vs. 23.4 ± 5.9 kg, p < 0.01). There were no differences between baseline and 6 months in time to complete 5 chair stands (9.9 ± 1.8 s vs. 10.4 ± 1.1 s, p = 0.37), SPPB score (11.5 ± 0.9 vs. 11.7 ± 0.6, p = 0.38), PROMIS-CAT score (46.8 ± 5.6 vs. 47.3 ± 8.6, p = 0.40) or 6-min walk distance (468 ± 86 m vs. 476 ± 105 m, p = 0.53).

**Table 1:** Muscle contractile function, fatigue resistance, and recovery measured by isokinetic dynamometry before and after aromatase inhibitor exposure.

	Baseline	6 months	P value
Torque-velocity test			
Maximal torque (nm/kg)	1.54 ± 0.31	1.53 ± 0.34	0.94
Maximal speed (rad/s)	12.2 ± 2.4	11.8 ± 1.1	0.62
Maximal power (w/kg)	3.45 ± 1.07	3.61 ± 0.79	0.44
Fatigue-recovery test			
Initial peak torque (nm/kg)	0.84 ± 0.17	0.84 ± 0.14	0.85
Final peak torque (nm/kg)	0.34 ± 0.06	0.32 ± 0.07	0.31
% Fatigue	58 ± 10	62 ± 10	0.42
Recovery peak torque (nm/kg)	0.83 ± 0.16	0.86 ± 0.15	0.34
Recovery half-life (s)	72 ± 38	62 ± 31	0.32

All data presented as mean ± SD. Contraction fatigue testing was performed at an angular velocity of 3.14 rad/s.

When exploring whether increase in RyR1 oxidation or loss of calstabin1 correlated with muscle function changes by dynamometry at the individual level, we found a significant correlation between change in oxidized RyR1 and maximal muscle power (r = 0.60, p = 0.02) and % fatigue (r = 0.57, p = 0.03). No correlations were found between bound calstabin1 and muscle function changes (Table 2). In addition, there was no correlation between change in grip strength and change in oxidized RyR1 (r = − 0.14, p = 0.65) or bound calstabin1 (r = 0.12, p = 0.69).

**Table 2:** Correlations between change in RyR1 biochemistry and muscle contractile properties (n = 14).

	Muscle function variable	Pearson correlation coefficient	P value
Change in oxidized RyR1	Maximal power	0.60	0.02
	% fatigue	0.58	0.03
Change in RyR1/bound calstabin1	Maximal power	0.22	0.44
	% fatigue	− 0.10	0.75

Corr., correlation; Coeff., coefficient.

### Body composition and bone density after AI exposure

There were no significant differences between baseline and 6 months in body fat, lean muscle, or bone density with 6 months of AI exposure (*see* Table 1*in supplemental data*).

### Serologic bone turnover markers after AI exposure

To determine whether muscle changes were correlated with early changes in bone turnover, we measured serum NTX-1 and TGF-β, both of which are increased in states of high bone turnover. We found 0.74 negative change in NTX-1 (p = 0.23) and 232 negative change in TGF-β (p = 0.64) from baseline to 6 months. When evaluating correlations between changes in bone turnover markers and change in RyR1 oxidation and loss of calstabin1 at the individual level, we found no significant associations (*see* Table 2*in supplemental data)*.

## Discussion

The skeletal complications of estrogen deprivation therapy in the treatment of breast cancer are long-term and well-established; in contrast, muscular complications are less well described and may have more immediate impact on physical function and quality of life. Based on compelling preclinical data that high bone turnover results in maladaptive changes in skeletal muscle and muscle weakness, we evaluated biochemical signatures and detailed clinical muscle function in women with breast cancer before and after AI therapy. As hypothesized, AI therapy resulted in a biochemical signature consistent with ‘leaky’ Ca^2+^ channels, oxidation of RyR1 and loss of its stabilizing unit calstabin1. In addition, while there were no differences in physical function after 6 months of AI exposure, oxidized RyR1 correlated with peak muscle power and rates of muscle fatigue.

Despite significant biochemical changes, there were no statistically significant changes in muscle function by dynamometry, SPPB, 6-min walk test, or self-reported functional health at the group level after 6 months of AI exposure. This is in contrast to a preclinical model of ovariectomized mice treated with AI versus placebo, which demonstrated a significant reduction in muscle-specific force of the extensor digitorum longus muscle in those treated with AI^3^. In our study, we did observe a significant decrease in grip strength at 6 months, consistent with another clinical observational report of women taking AIs^23^. However, this decrease in grip strength did not correlate with biochemical changes; this may be because decreases in grip strength have been primarily associated with joint pain in women on AIs, rather than reflective of actual muscle function^23,24^. Our study is the first to investigate muscle function in women on AIs with more comprehensive and specific endpoints beyond grip strength. Prior clinical observational studies have focused primarily on changes in muscle *mass* with AI exposure, with inconsistent results^25–28^. However, it is well-established that muscle mass is not a clear predictor of muscle function. Muscle function is a superior predictor of clinical outcomes including quality of life, activities of daily living, and functional independence, and is a significant independent predictor of mortality^29,30^. In a prospective study of older women with breast cancer, decline in physical function was associated with a 34% increased risk of death at 10 years^31^. Prior observational studies have found decline in physical function specifically in those patients experiencing AI-induced musculoskeletal syndrome, defined by presence of joint pain^24^. Increased joint pain has also been correlated with reduced physical activity levels, which in turn may lead to declines in physical function^32^. However, our study is limited by 6-month follow-up and many patients develop pain that may limit function at later time points; both of the observational studies referenced here evaluated patients several years into their AI therapy.

We did observe a correlation between molecular muscle changes and muscle power and fatigue resistance when evaluating changes in individual patients. The lack of significant changes in muscle function at the group level may be secondary to the limitations of small sample size or early evaluation of endpoints at 6 months. With only 6-month follow-up in our study, it is possible that we are seeing early biochemical changes in muscle associated with AI therapy that are still subclinical, and with longer follow-up, we may see more functional decline. Prior data analyzing skeletal complications associated with AI therapy suggest bone turnover starts rapidly as measured by serologic markers; however, it may be compensated and take significant time to cause additional systemic impact on muscle function that is visible at the clinical level^33^.

In this study, it is also possible that enrolling only a limited sample size of postmenopausal women at varying times after menopause impacted our ability to see muscle function changes. The gradual loss of estrogen with menopause and aging is associated with loss of muscle mass and function, likely related both to direct effects of estrogen signaling in muscle and a decline in physical activity with age. Hormone replacement therapy after menopause attenuates loss of muscle and may also improve muscle regeneration in response to exercise; thus, we would hypothesize that the complete estrogen deprivation with AI therapy would result in further decline in muscle function^34,35^. Future work analyzing muscular health in patients receiving endocrine therapy should include premenopausal women as many of those patients receive more rapid and severe estrogen decline with ovarian suppression and AI therapy, and may have more significant muscular impairment.

In addition, patients who had received chemotherapy were included in this analysis. Our prior work evaluated muscle power generation on a stationary bicycle following primary therapy for breast cancer, finding a similar reduction in power at 6 months in patients receiving endocrine therapy alone as those who received chemotherapy, without any evidence of recovery of function in either group by 12 months^36^. Based on this, we included patients regardless of prior chemotherapy and evaluated outcomes at 6 months in this analysis. However, this heterogeneity may have blunted baseline muscle function as preclinical work suggests cytotoxic chemotherapy induces protein degradation via activation of the NF-kB pathway and induction of the ubiquitin–proteasome system, while also reducing muscle protein synthesis^37,38^.

Preclinical work indicates that it may be possible to preserve RyR1 function in muscle. Inhibition of bone turnover with the bisphosphonate zolendronic acid results in reduced RyR1 oxidation, stabilization of calstabin1, and corresponding preservation of muscle function in mouse models^7^. In addition, mechanical signals delivered to bone either from exercise or vibrational stimulation can reduce bone turnover and improve muscle function in preclinical models of estrogen deprivation^39,40^. Translating these findings to clinic, we are currently studying the role of low intensity vibration to preserve muscle function in women receiving AIs who do not or cannot participate in regular exercise. In this ongoing trial (NCT03712813), we are collecting longitudinal changes in muscle architecture, function, and biochemistry over a 2 year period, and including both pre- and post-menopausal patients receiving estrogen deprivation.

To our knowledge, this study represents the only analysis of skeletal muscle tissue in the most common population of breast cancer patients. Repeated muscle biopsies were feasible and safe in this population, with only one of 15 patients declining a follow-up biopsy and no adverse events reported. This is a comprehensive assessment across molecular, subjective, and objective muscle function measures in a population where this was not previously explored, and is based on a mechanistic hypothesis that could be therapeutically exploited to improve patient outcomes. While we observed changes in RyR1, there may be other biochemical markers of muscle impairment associated with endocrine therapy worthy of investigation, and future work should more comprehensively analyze muscle tissue in these patients. Musculoskeletal complications are a major source of morbidity due to breast cancer and its therapy; however, little has been done to explore the ‘musculo’ aspect of musculoskeletal health in survivors. This may have significant implications for short- and long-term quality of life, medication adherence, physical activity participation, and physical function. More comprehensive assessments of *musculo*skeletal health are possible and needed across the cancer continuum.

### Supplementary Information

Supplementary Information.

## Data Availability

The data generated in this study are available upon request from the corresponding author.
