# Supplementary material for: Molecular and clinical effects of aromatase inhibitor therapy on skeletal muscle function in early-stage breast cancer
Source: Sci Rep. 2024 Jan 10;14:1029. doi: 10.1038/s41598-024-51751-y (PMC10781701; doi:10.1038/s41598-024-51751-y)
Supplement: Supplementary file 1 — Supplementary Information. [file 41598_2024_51751_MOESM1_ESM.pdf]

Supplemental Materials

Molecular and clinical effects of aromatase inhibitor therapy on skeletal muscle function in early-stage breast cancer

Seibert et al.

Supplemental Table 1. DXA body composition and bone density measures

	BASELINE	6 MONTHS	P VALUE
**BODY COMPOSITION MEASURES**			
BMI (kg/m^2)	31.7 ± 5.4	30.9 ± 5.0	0.18
Total body fat (%)	43.4 ± 6.0	42.9 ± 5.2	0.53
Total lean mass (g)	42007 ± 6036	41173 ± 5502	0.12
Total fat mass (g)	34855 ± 8594	33404 ± 7563	0.14
**BONE DENSITY MEASURES**			
Femoral neck BMD	0.92 ± 0.14	0.91 ± 0.11	0.95
Femoral neck T-score	-0.67 ± 0.89	-0.74 ± 0.75	0.50
Lumbar spine BMD	1.13 ± 0.18	1.13 ± 0.19	0.86
Lumbar spine T-score	0.12 ± 1.00	0.04 ± 1.18	0.89

Supplemental Table 2. Correlations between change in RyR1 biochemistry and bone turnover markers (n=14).

	SEROLOGIC BONE TURNOVER MARKER	PEARSON CORRELATION COEFFICIENT	P VALUE
**CHANGE IN OXIDIZED RYR1**	NTx<br>TGF-β	-0.01<br>-0.02	0.98<br>0.94
**CHANGE IN RYR1/BOUND CALSTABIN1**	NTx<br>TGF-β	0.02<br>0.29	0.93<br>0.32

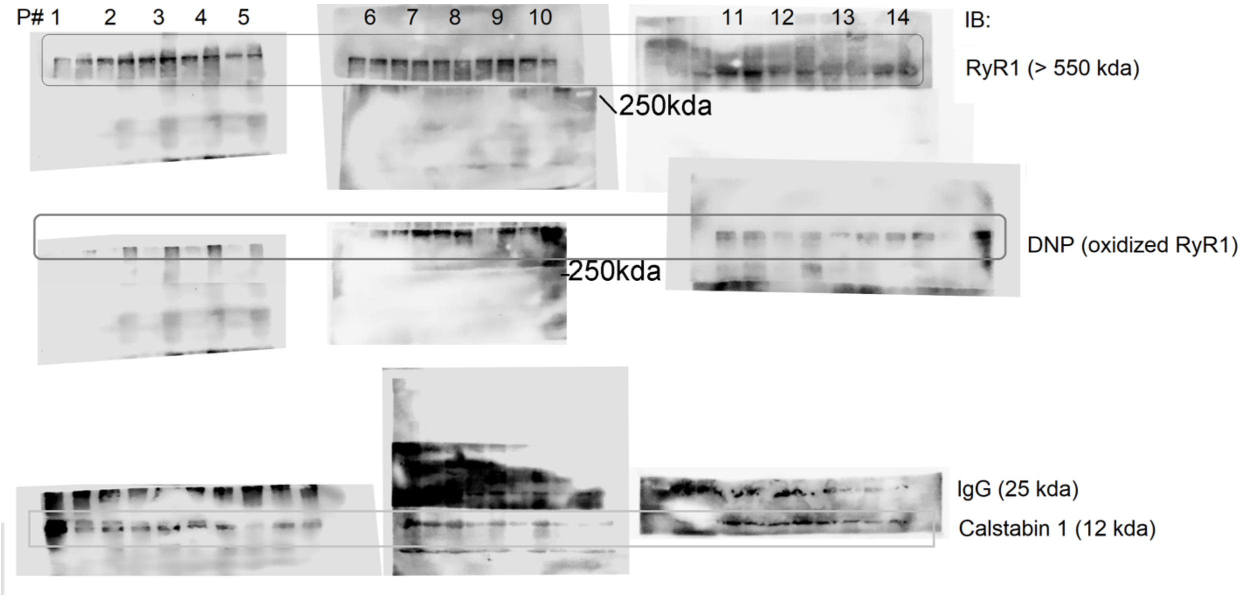

Supplemental Figure 1: Original uncropped Western blot gel for detecting RyR1, DNP (oxidized RyR1) and Calstabin 1 signals. Boxes indicate the cropped regions in Extended Data Fig. 2a. Lanes 1 to 14 correspond to samples from patient #1 to #14, before (left) and after 6-month treatment (right). A long-exposure blot gel showing the oxidation status of RyR1 (second run using the same cell samples as top panel). The Oxidized Protein Western Blot Kit was used, whereby carbonyl groups of immunoprecipitated RyR1 were derivatized to 2,4 dinitrophenylhydrazone (DNP) by reaction with 2,4 dinitrophenylhydrazine.
